# Perception of direct gaze in a video-conference setting: the effects of position and size

**DOI:** 10.1186/s41235-022-00418-1

**Published:** 2022-07-22

**Authors:** Gernot Horstmann, Linda Linke

**Affiliations:** 1 Department of Psychology, Bielefeld University, Bielefeld, Germany; 2 CITEC - Center for Cognitive Interaction Technology, Bielefeld University, Bielefeld, Germany

**Keywords:** Gaze, Gaze perception, Video conference, Perception of being looked at

## Abstract

A common problem in video conferences is gaze direction. In face-to-face communication, it is common that speaker and listener intermittently look at each other. In a video-conference setting, where multiple participants are on the screen, things are complicated and not necessarily optimal. If the listener feels looked at when the speaker looks into the camera, how tolerant is the listener for slight deviations? And does this depend on the position of the speaker’s tile on the screen, or the size of the tile? In a first experiment, participants from a student population judged whether they are looked at, while vertical gaze direction of the looker was varied. Furthermore, the position of the tile on the screen varied. The results showed that a slightly upward directed gaze was optimal for the direct gaze judgment, with a width of ± 4 degrees. Optimal gaze direction was somewhat higher for tiles at the bottom of the screen. A second experiment tested the effect of size on the perception of horizontal gaze directions. Size was found to increase the gaze cone. The paper concludes with some recommendations for a setup of video conference systems, optimized for perceived gaze contact.

## Significance

Gaze is an important social cue not at least because gaze direction is tightly coupled with attention. Because of its ubiquity, it is probably adequate to call gaze the most influential tool of mind reading, because what we are looking at is most of the time of interest for us and the basis for cognition, emotion, and action. Human-human interactions are a particular field of gaze perception, and in the case of direct gaze, the looker’s object is the face of another person. Nowadays, quite some share of communication is done with the help of media, and in particular video conferencing applications. In face to face communication, speakers look at each other, at least intermittedly. In a video conference setting, this is difficult or restricted, and the present research contributes to an assessment and a discussion of the conditions of direct gaze in this setting.

## Introduction

Video conferences have become an everyday activity in recent years. For the present context, a video conference is conceived of as a substitute for an in-person meeting between two or more participants, usually using a video camera and a screen (often integrated in a laptop computer, a tablet, or a smartphone) for each participant, and a software application that transmits the video stream via the internet, distributes them among the participants, and organizes the visual presentation of the streams on the participants’ screens.

A common problem within this setting is gaze direction. In general, gaze direction is determined by the ongoing and dynamically changing aspects of the looker’s task (Schneider et al., 2013). It is dominated by the current needs for visual high-detail information, when the high-resolution area of the human retina, the fovea, is aligned with the sought-for visual input (for an overview, see Tatler et al., 2011).

Although looking behavior is primarily instrumental for the looker, it is additionally also communicative (Kendon & Cook, 1969). Importantly, eye movements are freely visible to observers, and highly correlated with a quality in which the observer is interested in: the focusing of the mind’s eye. Due to the high correlation between observable eye movements (the signifier) and the part of the world in that the looker currently concerns, (the signified), they fulfill conditions for evolved signals in an animal signaling perspective (Maynard-Smith & Harper, 1995). Some features of the eye seem even to have evolved mainly for communication (Kobayashi & Kohshima, 2001 ): The sclera of the human eye is especially large and light and has good contrast to the pigmented iris and the enclosed dark pupil. This stimulus is rare among mammals and in particular, primates, where only bonobos have a relatively light sclera (even though not as bright as the human sclera, cf. Perea-García et al., 2019).

An important physical aspect of gaze direction is the rotation of the eyeball. The rotation of the eyeball leads to lawful changes in the positioning of the darker iris-pupil complex within the lighter sclera. Previous studies have investigated the sensitivity of perceiving gaze direction from eyeball rotation. These studies can be mainly divided into two major tasks: Dyadic or triadic gaze tasks. In the dyadic task (e.g. Gibson & Pick, 1963), the question of being looked at is addressed, which is often also termed as direct gaze in the literature. While the looker’s eye position serves as the stimulus, the observer must judge if he is the target of the looker’s gaze. This interaction between looker and observer is dyadic. In triadic gaze tasks (e.g. Anstis et al., 1969; West, 2015), the setting involves the looker, the observer, and a separate (“third”) object. Here, again the looker’s eye position serves as the stimulus, but now the observer must judge if the looker’s gaze is directed to a separate (“third”) object and not himself.

We will focus on dyadic tasks here after noticing that people’s triadic judgements of gaze direction show very good sensitivity to eyeball rotations, with an accuracy of about 1° of eyeball rotation for a distance of 1 m (Symons et al., 2004). In other words, a looker-observer distance of 1 m, the observer sees when the looker shifts his gaze from the inner to the outer corner of one eye. Despite this accuracy, the observer tends to overestimate lateral gaze position by a factor between 1.2 and 2.0 depending on conditions that are not fully understood yet (e.g., Anstis et al., 1969; Masame, 1990; West, 2011). Dyadic judgement tasks have consistently shown that there is a range of gaze directions that is accepted by the observer as targeted to him. These range of gaze directions result in an area—rather than a point—of direct gaze. Gamer and Hecht (2007) described this area as the gaze cone. The cone metaphor is implied by a constant cone angle size (Gamer & Hecht, 2007; see also Horstmann & Linke, 2021). The gaze cone size has been measured to be 5–15°, somewhat depending on the task and on specifics of the measuring situation. Clifford and colleagues (Balsdon & Clifford, 2018; Mareschal et al., 2014) have argued that there is a bias to perceive direct gaze and found the gaze cone to be larger with perceptual uncertainty (but see Hecht et al., 2014). Hecht et al. (2014) induced simulated vision impairments and found that the influence of head rotations increased. They also report more variable judgements with elderly participants compared to the younger participants, without an effect on cone size. Individual differences in gaze cone size have been reported by Lobmaier et al. (2021), and ostracism seems to increase gaze cone size (Lyyra et al., 2017). Additionally, Gamer et al. (2011) report that the gaze cone width can be influenced by social phobia (see also, Harbort et al., 2013).

**Fig. 1:**
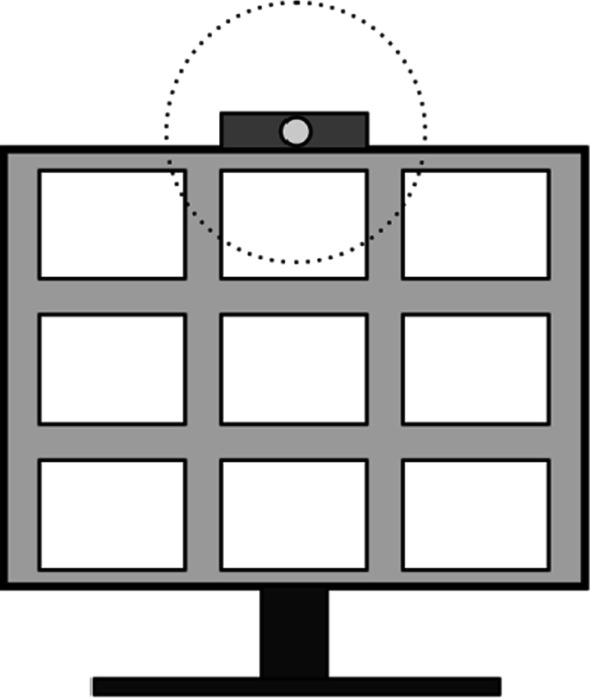
In a setting with 9 tiles on a screen and a top-mounted camera, the listener will feel looked at when the speaker looks directly into the camera or fixates at least within an area that corresponds to the gaze cone width (dotted circle). This often includes only the tile in the top row immediately under the camera

A specific variant of a dyadic judgment task is a video conference setting, in which observers judge the gaze of a looker in a video transmission. Previous studies have explored the value of gaze awareness in remote collaborations involving stationary tasks performed on a computer screen (Brennan et al., 2008; Qvarfordt & Zhai, 2005), or physical tasks involving limited mobility (Akkil et al., 2016; Gupta et al., 2016). The results indicate that gaze awareness makes collaboration easier by allowing effortless reference to spatial information and by contributing to an improved feeling of presence. Moreover, there is also a well-documented tendency to follow gaze automatically and to shift attention into the direction indicated by the eyes of an observed face (see also Dalmaso et al., 2020, for a review of the effect of gaze on attention). Importantly, research on gaze direction and the gaze cone has implications on video-conference settings in that listeners will often not feel being looked at although the speaker is actually looking straight at the picture of the listener. Assuming a gaze cone of 10 degrees in diameter and a typical distance between the participant and the screen of 57 cm, this means, that the actual target of the speaker’s gaze must be within a 5 cm radius of the camera (see Fig. 1, for illustration). In a setting, where nine participants of a video meeting are displayed on a desktop computer screen in a 3 × 3 matrix, this means that when the speaker looks at the top tile of the central column of tiles, listeners probably have the perception of being looked at (assuming that the camera is positioned at the top of the screen). Correspondingly, if the speaker looks at one of the remaining eight tiles, listeners have the perception of not being looked at. The same applies, of course, to the listeners: If the speaker’s tile is positioned at the top position just below the camera, and the listener is looking at this position, the speaker will have the perception of being looked at, but less so at the other positions. Yet, there is one caveat. The above discussion assumes a circular and symmetrical cone. Contrary to this simple and intuitive idea, one study (Chen, 2002) found an asymmetrical cone, with a very small radius in the upper hemifield, and a large radius in the lower hemifield. While this seems to be a rather atypical result within the literature, the report has received considerable attention in the more technically leaned video-conference literature and has been taken as an argument that the camera should be mounted on top of the monitor (as it almost always is). The argument is that the listener tolerates downward directed gaze as direct and thus often feels being looked at when the looker is filmed by a top mounted camera.

A related problem is picture size. In a two-person video conference meeting, the picture of the other person may be live sized on a desktop computer screen, and somewhat smaller on a notebook computer or tablet screen. In many-person meetings, however, a tile display is usually used, and miniature pictures are presented in a way that many participants are visible on the same screen simultaneously. Picture size may have its own effects. In particular, a size reduction necessarily leads to a resolution reduction both on the screen and on the retina. More generally it can be said that reducing picture size reduces information and leads to higher uncertainty about gaze direction. It has been argued that uncertainty leads to a generalized tendency toward direct gaze perceptions (Mareschal et al., 2014).

A final point relevant to the analysis of a video conference meeting is the Mona Lisa effect. (Hecht et al., 2014; Horstmann & Loth, 2019; Todorovic, 2006). The Mona Lisa effect is a constancy phenomenon for a 2-D representation (i.e., a flat picture) of a straight looking person. It entails that the perception of being looked at when viewing the picture is largely independent of the viewpoint of the observer relative to the picture. That is, changes in distance, slant, or rotation of the picture should not lead to a change in the perception of being looked at. Obviously, because of the Mona Lisa effect, the positioning of a tile on the monitor or its size should make no difference with respect to perceived gaze direction.

The present research addresses the problems of position and picture size. Experiment 1 focuses on position, while additionally varying size. Experiment 2 holds constant position and examines size in more detail. Both experiments use the method of constant stimuli to infer the principal direction of gaze and the size of the gaze cone. The downward bias hypothesis predicts that observers have an asymmetrical gaze cone vertically, and that gaze cone radius is larger in the lower field of gaze. The uncertainty hypothesis predicts that the gaze cone is generally wider with smaller pictures.

## Experiment 1

Experiment 1 probes the distribution of vertical gaze directions that are perceived as directed to the observer. A particular question is whether the gaze cone is more or less symmetrical vertically, or larger in the lower gaze positions. In addition, picture size and picture position are varied. Size and position are both variables relevant in a video conference setting, and it is often assumed that they have no effect on the perception of being looked at, because of the Mona Lisa effect.

### Methods

#### Participants

Sixteen participants, 9 women and 7 men, between 19 and 28 years of age (median = 23.5) participated in the one-hour experiment. One participant was lost (see below), reducing the sample size to 15. The sample size was chosen on the assumption of a large effect size. In within designs, large effect sizes can be obtained by minimizing measurement errors through a high number of measurement repetitions. The present sample size is adequate to yield an effect with a size of *d* = 0.66 (test against zero), when alpha is set to 0.05 and power to 0.80. The calculation was done using the R-package Webpower (Zhang & Yuan, 2018). Participants had normal acuity and passed a color deficiency test. They received course credit or candy for their participation, and they gave written informed consent before participation; the experiments were approved by Bielefeld University’s ethics committee and complied with the ethical guidelines of the German Psychological Association (DGPs), and with the provisions of the World Medical Association Declaration of Helsinki.

#### Stimuli

**Fig. 2:**
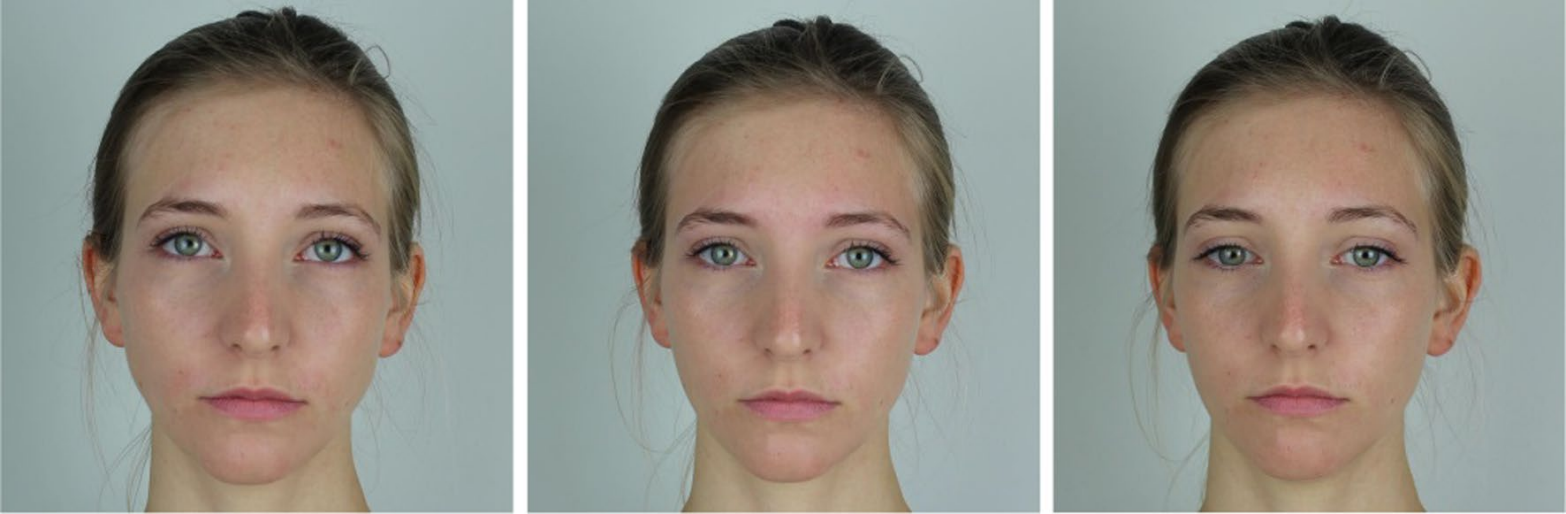
Three examples for the looker model in Experiment 1. Left: 9° upward; middle: gaze straight at the camera; right: 12° downward gaze

Stimuli were obtained with a Canon EOS 600D with a resolution of 3120*2080, which was positioned between two semi-professional softbox photography lights. The looker was a 23-year-old woman with green eyes (see Fig. 2 for examples). The camera was positioned at eye level of the looker with a nose-lens distance of 57 cm. The central fixation point was at the center of the lens, and the first two fixation points above and below the center were on the respective fringes of the lens above and below the center. A cardboard with a circular opening fitted around the lens carried the remaining fixation points. All fixation points were separated by half the radius of the lens, each subtending 3 degrees of visual angle. The photographs were then cropped to a tile format (16:9) and resized. Two sizes of each stimulus were used, one 1600 × 900 pixels (23 × 22 cm) and one 640 × 360 pixels (17*9.5 cm).

#### Apparatus

Stimuli were presented on a Samsung SyncMaster171P (34 × 27 cm) with a resolution of 1280*1024. The monitor was connected to a Linux-operated (Ubuntu 16.04 LTS) workstation. Stimulus presentation and event scheduling were controlled by a Python script using the PsychoPy package (Pierce, 2007, 2009). Responses were collected as key presses on a regular USB-keyboard. A chin rest was positioned 57 cm in front of the monitor. A height-adjustable chair was used to position each participant to the same height, at which the eyes are level with the center of the screen. At this distance, effective screen resolution was 46 px/degree of visual angle.

#### Design

In the first experiment, the picture size (large vs. small) and the elevation (up vs. down) of the picture position were varied. The picture’s vertical position was changed such that the upper or the lower border almost touched the screen border, which was plus or minus 100 pixels for the large pictures and plus or minus 300 pixels for the small. Eight gaze directions were included, ranging from 9 to − 12 degrees in 3 degrees steps. Each of the 8 × 4 = 32 cells was presented 20 times (640 trials in total). The raw dependent variable was the judgment of being looked at (yes/no). For each of the four combinations of picture size and picture position, a Gaussian was fitted to the raw data (proportion of yes answers for the given gaze directions) to obtain the central tendency and the width of the Gaussian. These derived parameters for the Gaussian were the actual dependent variables.

#### Procedure

Each trial started with the presentation of a fixation circle for one second, followed by the target display. The target display stayed on until the participant signaled his or her judgement by pressing the respective key. Next, the target display disappeared and was replaced by a word (yes / no) indicating the meaning of the given response. Then the next trial began.

### Results and discussion

Table 1 shows the mean proportion of “yes” answers for each of the gaze positions and the four conditions (2 sizes and 2 positions).

**Table 1:** Means and standard deviations for the proportion of “yes” answers as a function of a 2 (size) × 2 (position) × 8 (direction). The means have been set in bold to increase readability

	Direction															
	− 12		− 9		− 6		− 3		0		3		6		9	
Position	*M*	SD	*M*	SD	*M*	SD	*M*	SD	*M*	SD	*M*	SD	*M*	SD	*M*	SD
*Small*																
Down	0.00	0.00	0.02	0.04	0.34	0.32	0.45	0.32	0.91	0.13	0.94	0.08	0.55	0.35	0.07	0.14
Up	0.11	0.22	0.16	0.25	0.46	0.40	0.61	0.38	0.95	0.09	0.84	0.12	0.27	0.27	0.02	0.04
*Large*																
Down	0.02	0.03	0.04	0.06	0.33	0.29	0.43	0.37	0.92	0.11	0.97	0.07	0.58	0.32	0.07	0.12
Up	0.05	0.08	0.16	0.27	0.47	0.40	0.50	0.41	0.94	0.10	0.97	0.07	0.46	0.30	0.05	0.08

*M* and *SD* represent mean and standard deviation, respectively

Proportion correct was fitted to a Gaussian for each participant and each of the four conditions, using the R-package *quickpsy* (Linares & López-Moliner, 2016). The resulting fits were screened for outliers with respect to the center and to the width of the Gaussian, using the 98th quantile as the cutoff, resulting in the exclusion of one participant, and reducing the sample size to 15.

**Fig. 3:**
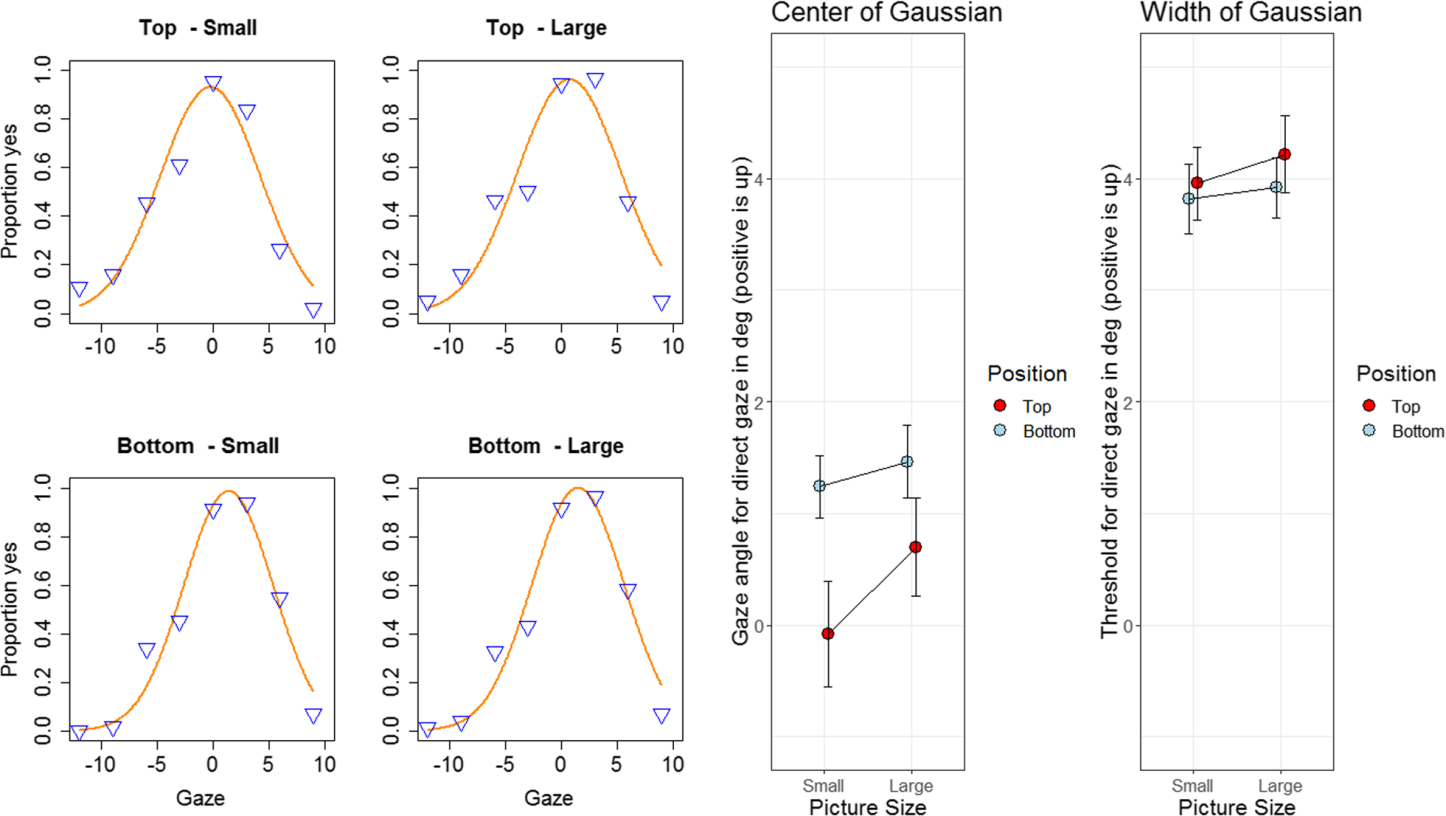
Left: the grand mean data points and a graphical depiction of the fitted Gaussians. Middle: center of the Gaussian, separately for each combination of picture position and picture size. Right: width of the Gaussian, separately for each combination of picture position and picture size

An ANOVA (all reported statistical tests used procedures for within comparisons) of the mean of the Gaussian with the variables picture size and picture position rendered significant main effects for size, *F* (1,14) = 11.49, *p* = 0.004, eta^2^ = 0.03, position, *F* (1,14) = 12.72, *p* = 0.003, eta^2^ = 0.11, and the Size × Position interaction, *F* (1, 14) = 5.48, *p* = 0.03, eta^2^ = 0.01. The center parameter was higher with large than with small pictures (1.1 vs. 0.6), and higher for the bottom than for the top positions (1.5 vs. 0.3). See also Fig. 3. The interaction reflects that the difference in the position effect was larger with the small pictures. Follow-up tests revealed that the position effect was significant in the small picture condition, *t* (14) = 3.44, *p* = 0.004, *d* = 0.81, and also in the large picture condition, *t* (14) = 3.33, *p* = 0.005, *d* = 0.45.

There was thus a robust effect of stimulus position, with lower positions requiring more upward rotated eyes than higher positions for the perception of being looked at. This result runs counter a strong version of the Mona Lisa effect which would predict no effect of position on perceived gaze direction at all. That the position effect was found for the large pictures as well as for the small pictures is somewhat surprising given that the positions shifted only slightly. Gaze perception apparently is quite sensitive to position changes.

The analysis of the width of the Gaussian rendered a stimulus position effect only, *F* (1, 14) = 8.09, *p* = 0.013, eta^2^ = 0.01, indicating a somewhat larger width when the picture was displayed at the top (4.1°) rather than the bottom (3.9°) position of the screen (other *F*s < 1.88, *p*s > 0.19).Fig. 2 displays the means. The standard deviation of the Gaussian was around 4 degrees, indicating a gaze cone of 8 degrees in the vertical diameter. It was significantly wider at the top position, but the numerical difference was minute.

**Fig. 4:**
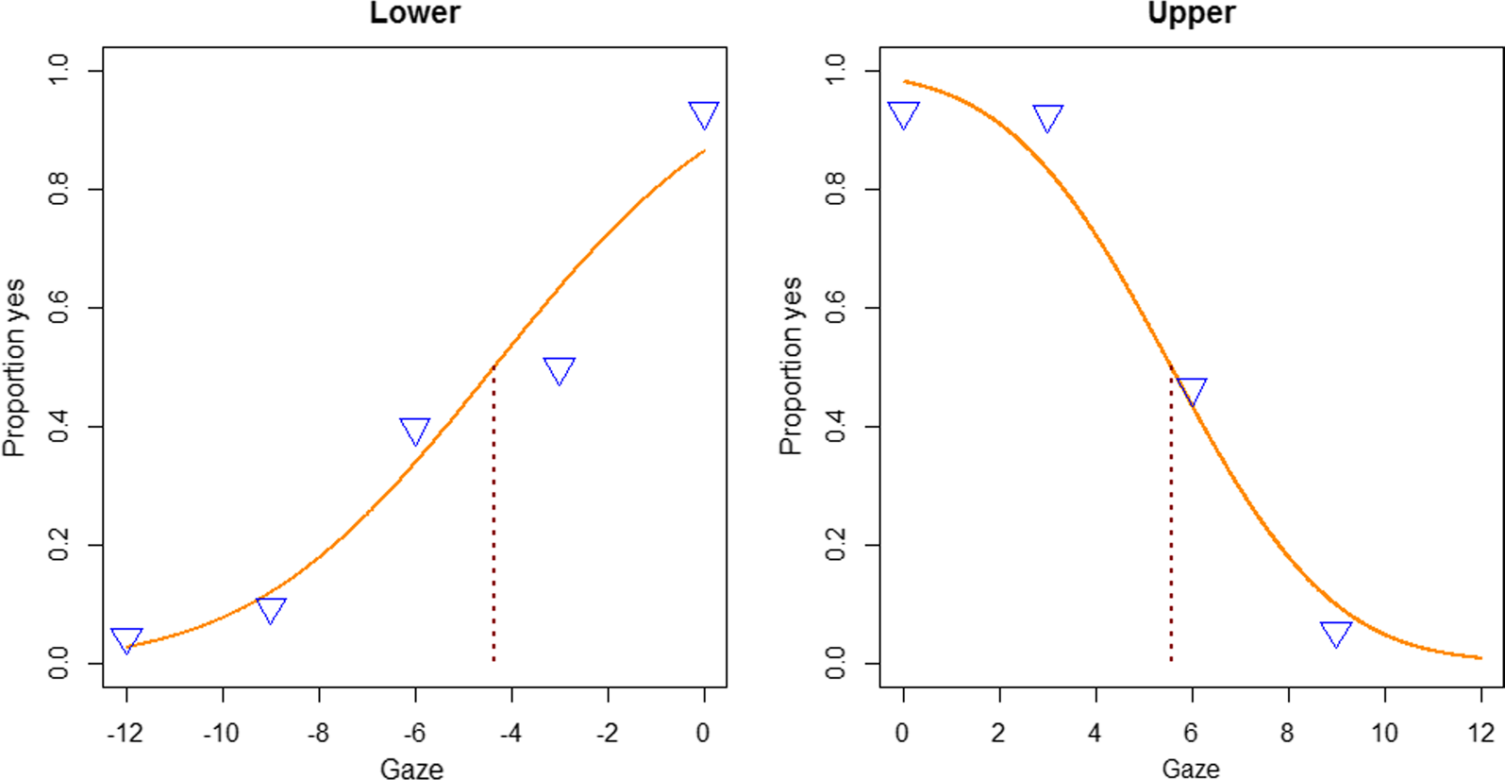
Separate fittings of the lower and the upper half with cumulative Gaussians, with measurements collapsed over the factors size and position

The data show no obvious trend toward a higher threshold for downward than for upward directed gaze. To more formally test a possible downward bias, the range of objective gaze directions was split into halves at zero. (Note that the zero is included because it provides a point near the maximums, which is preferable to obtain a reasonable fit; see Fig. 4). For further analysis, the upward directed distribution was inverted such that the inverted proportion correct was 1 minus proportion correct. The downward and the (inverted) upward distribution were then fitted to a cumulative Gaussian to extract the threshold (*p* = 0.5) for downward and upward directed gaze, respectively. The obtained thresholds were then analyzed by a hemifield (lower vs. upper) × size (small vs. large) × position (top vs. bottom) ANOVA. The ANOVA revealed a main effect for position, *F* (1,14) = 5.62, *p* = 0.033 eta^2^ = 0.005, and a just not significant main effect for hemifield, *F* (1,14) = 4.44, *p* = 0.054, eta^2^ = 0.098 (main effect for size, *F* < 1). Note that the just not significant effect is in the “wrong” direction, and that the threshold was in fact somewhat larger in the upper hemifield. The main effects were modified by significant interactions: Hemifield × Size, *F* (1,14) = 11.69, *p* = 0.004, *eta*^*2*^ = 0.015, Hemifield × Position, *F* (1,14) = 11.02, *p* = 0.005, *eta*^*2*^ = 0.047, and Hemifield × Position × Size, *F* (1,14) = 7.88, *p* = 0.013, *eta*^*2*^ = 0.006 (the remaining Size × Position interaction was not significant, *F* < 1). The threshold for the lower side was smaller than for the upper side, but this effect was more pronounced for the large pictures (3.91 vs. 5.86) than for the small pictures (4.25 vs. 5.13), and it was also more pronounced for the bottom position (3.21 vs. 5.67) than for the upper position (4.21 vs. 5.19). Post hoc t-tests revealed that the gaze direction effect was significant for the large pictures in the lower position, *t*(14) = 4.07, *p* = 0.001, *d* = 1.4, and for the small pictures in the lower position, *t*(14) = 4.25, *p* < 0.001, *d* = 1.1, but for neither comparison in the upper position, *t*s < 1.56, *p*s > 0.14.

The analyses did not find a downward bias. Contrary to that, there was a slight upward bias in the sense that the upper half of gaze directions was slightly wider than the lower half; this effect was larger for the bottom position, and actually, only statistically significant only for this position. In addition, gaze direction was on average perceived as direct when the eyes were rotated slightly upward. This tendency was pronounced for the lower picture positions and almost absent for the upper picture position. There is no indication in the data that the lower extension of the gaze cone is wider than the other extensions. In contrast, the upper extension seems to be larger.

## Experiment 2

With Experiment 2, we took a closer look at the effect of picture size. Picture size interacted with gaze direction in Experiment 1, where the position effect was larger with smaller pictures. There was, however, no significant effect of size on the width of the cone. Such an effect, however, is expected on the assumption that (a) reducing size reduces the amount of available information about eye position, which in turn (b) increases the uncertainty for the observer. As observers are biased to assume directed gaze if in doubt (Mareschal et al., 2014), the gaze cone should be larger for smaller pictures.

It is possible that the effect of size was overshadowed by other effects, or that the effect of size is weak for vertical gaze directions. Experiment 2 targets the predicted effect of size on cone width using picture size as the single manipulation. Size was manipulated in three levels: full size (i.e., the size of a real head), half size, and quarter size, which also increased the range of tested sizes relative to Experiment 1. Experiment 2 tested horizontal gaze positions. Horizontal gaze positions were used because these are simpler than vertical gaze positions, where the eye shape changes with gaze direction due to the movement of the upper lid. An additional size was included, and the face was presented in full size, half size, and quarter size.

### Methods

#### Participants

Thirteen participants, 5 men and 7 woman (one person preferred not to choose between male and female), between 19 and 63 years of age (median = 26) participated in the half-hour experiment. One participant was lost (see below), reducing the sample to 12 participants. This sample size was adequate to find an effect of *d* = 0.76 larger than zero with an alpha = 0.05 and a power of 0.80. Participants had normal acuity and passed a color deficiency test. The participants received course credit or candy for their participation, and they gave written informed consent before participation; the experiments were approved by Bielefeld University’s ethics committee and complied with the ethical guidelines of the German Psychological Association (DGPs), and with the provisions of the World Medical Association Declaration of Helsinki.

#### Stimuli

Stimuli were obtained with a Canon 6D (20 megapixels) equipped with a 70–200 mm f2.8 lens. The pictures were shot at approximately 120 mm focal length and a resolution of 3648*5472 pixels. The camera was flanked by semi-professional lightning which was positioned to the aim of a levelled lightning of the face (approximately 600 Watts on each side). The looker model was a young man with brown eyes. The camera was positioned at eye level of the looker model with a nose-lens distance of 162 cm. Using this distance is suggested by an argument presented by Horstmann and Linke (2021), being that vergence is irrelevant at this and larger distance because the observer cannot furthermore distinguish between the correct vergence at this distance, and less or even no vergence. The central fixation point was at the center of the lens. A stripe of cardboard with a circular opening fitted around the lens carried the remaining fixation points, which were 10 cm apart from each other. The eccentricities of the fixation points were − 10.5, − 7.0, − 3.5, 0, 3.5, 7.0, and 10.5°, from left to right. Cropping and resizing of the picture was controlled by a custom Python script using the image manipulation library PIL. The pictures were cropped to 980*1024 pixels, with all pictures being centered on the nose root equidistant to both eyes. Pictures of all sizes were centered on the screen. The full size picture had an interpupil distance of 189 pixels (half and quarter size the corresponding fractions). In the laboratory, this corresponded to an interpupil distance of 5.8 cm for the full size picture.

#### Apparatus

Stimuli were presented on a Samsung SyncMaster171P (34 × 27 cm) with a resolution of 1280*1024. The monitor was connected to a Linux-operated (Ubuntu 16.04 LTS) workstation. Stimulus presentation and event scheduling were controlled by a Python script using the PsychoPy package (Pierce, 2007, 2009). Responses were collected as key presses on a regular USB-keyboard. A chin rest was positioned 162 cm in front of the monitor. A height-adjustable chair was used to position each participant to the same height, at which the eyes are level with the center of the screen. Thus, effective screen resolution was 131 px/degree of visual angle. Note that even though many displays used in a video conference setting have a higher resolution than our laboratory computer display, the larger viewing distance in this experiment counters a possible effect of lower resolution and yields an effective screen resolution similar to a present office computer.

#### Design

The main experimental variable was picture size (full vs. half vs. quarter). At each size, the gaze direction of the looker was varied in 7 steps. Each of the 7 × 3 = 21 cells was presented 22 times (462 trials in total). The raw dependent variable was the judgment of being looked at (yes/no). For each of the four combinations of picture size and picture position, a Gaussian was fitted to the raw data (proportion of yes answers for the given gaze directions) to obtain the central tendency and the width of the Gaussian. These derived parameters for the Gaussian were the actual dependent variables.

#### Procedure

Each trial began with the presentation of a fixation circle for one second, followed by the target display. The target display stayed on until the participant signaled his or her judgement by pressing the respective key. Then the target display disappeared and was replaced by a word (yes/no) indicating the meaning of the given response. Then the next trial began.

### Results and discussion

Proportion correct was fitted to a Gaussian for each participant and each of the three conditions, using the R-package *quickpsy* (Linares & López-Moliner, 2016). The resulting fits were screened for outliers with respect to the center and to the width of the Gaussian, using the 98th quantile as the cutoff, resulting in the exclusion of one participant, and reducing the sample size to 12.

The main analysis was an ANOVA of the width parameters with the variable size (full vs. half vs. quarter), which revealed a significant main effect of size, *F* (1,11) = 7.03, *p* = 0.021, eta^2^ = 0.39. The width of the Gaussian increased as the picture sizes decreased (2.63, 2.80, 3.00, for full, half, and quarter size, respectively).

An additional ANOVA of the center parameters with the variable size (full vs. half vs. quarter) also revealed a significant main effect, *F* (1,11) = 25.08, *p* < 0.001, eta^2^ = 0.70. The center shifted somewhat to the right with smaller pictures (0.11, 1.34, 1.44, for full, half, and quarter size, respectively).

To summarize, we found that smaller pictures yielded a wider gaze cone than larger pictures, which is predicted by Mareschal et al.’s (2014) uncertainty hypothesis. An unexpected result was a small shift of the gaze to the right of around 1° with the smaller pictures. There is no obvious explanation. Although effects are known in the literature to explain apparent shifts of gaze, for example, because of a bloodshot (Ando & Osaka, 1998), or uneven illumination (West, 2013), it is not clear why this shift depended also on picture size.

## General discussion

The aim of this study was to examine the perception of being looked at under conditions that typically occur in a video-conference setting, with special foci on a possible downward bias and a possible effect of picture size. Experiment 1 revealed a vertical gaze cone diameter of 8 degrees of visual angle in diameter for a video-conference setting. The center of the gaze cone was slightly upward, an effect that was somewhat stronger when the picture was presented at the bottom than at the top of the screen, defying a strong version of the Mona Lisa effect. An examination of the upper and the lower half of the cone did not reveal a downward bias; in contrast, the upper half was actually larger than the lower half, although not significantly. While Experiment 1 did not find an effect of picture size on the width of the cone for vertical gaze positions, Experiment 2 revealed a widening of the gaze cone for horizontal gaze positions when picture size was reduced. Depending on size, the horizontal gaze cone diameter varied between 5.2 and 6°.

Overall, a clear downward bias was not obtained, and in fact, the results of Experiment 1 actually point in the opposite direction. The average gaze direction (indicated by the center parameter of the Gaussian) was slightly in the positive, indicating that gaze had to be directed somewhat upward to be perceived as straight, or put otherwise, that the gaze was perceived as somewhat lower than it actually was. Also, the upper half of the gaze cone was somewhat larger when the gaze directions were split at the objective straight gaze, in particular, for the lower positions (this was not significant for the upper positions). Note that the effects on the center parameter of the Gaussian and the width parameter of the cumulative Gaussian are plausibly the same effect that registers in two alternative ways of analysis, with the latter analysis being better suited to test the downward bias hypothesis.

According to our findings, the cone is approximately symmetrical vertically and of circular or oval shape. The present cone size and shape is consistent with older research from the Bell Laboratories from 1969, reported in Chen (2002), which found thresholds of 4.5° horizontal and 5.5° vertical for losing eye contact in a video-conference setting. It differs, however, from Chen’s own study, which reports high thresholds (i.e., small degrees of visual angle) to the left, right, and top, and a low threshold below the camera, resulting in an asymmetrical shape with its center below the camera. Unfortunately, the conditions are not all optimally reported by Chen (2002), and it is possible that the low resolution of the pictures that seemed to be presented at a considerable distance (2.4 m) contribute to the large vertical downward extension of the gaze cone. It might be noted that the present results are also more in line with Anstis et al. (1969) than Chen’s (2002) results: Anstis et al. (1969) found high accuracy in triadic judgements for the vertical dimension, without an asymmetry above or below the horizontal axis. On the basis of his results, Chen advocated a top camera, and his study is often cited for this claim. Obviously, the present results are at odds with this rationale for this recommendation.

Somewhat unexpectedly, and contrary to the assumed truism of a Mona Lisa effect, picture position had an effect on direct gaze perception. Put simply, faces at the bottom position needed to look somewhat upward to be perceived as straight, which is not the case for pictures at the top position. One might argue though that the Mona Lisa effect concerns a picture at a fixed position and observers at different positions relative to the picture, while in the present research, the observer’s position was fixed, while the position of the pictures changed. However, it seems common and reasonable to lump together these two situations, as the commonality between these two is the change of the relative positions of picture and observer.

The effect of picture size (found in Experiment 2) on the width of the gaze cone confirms the uncertainty hypothesis that uncertainty leads to a bias to assume directed gaze. The bias may be rooted in evolutionary factors as direct gaze is a signal for positive or negative (in opposition to neutral) outcomes, and a false alarm is evolutionary often less costly than a miss. There are also practical implications. Assuming that the impression of being looked at is a “good” feature in a video-conference setting, small face sizes could be argued to be desirable. One wonders, however, whether individual factors might alter this bias, in particular social anxiety that have already been shown to widen the gaze cone.

A limitation of the present study is that gaze direction was manipulated only vertically and horizontally, without a manipulation of diagonally varying gaze. Two scenarios are conceivable. If horizontal and vertical gaze are independent from each other (implying that the cross section of the gaze cone has the shape of a rectangle), the diagonal diameter would be somewhat larger than the vertical and horizontal diameter. According to the gaze cone concept, however, the diagonal would be between the horizontal and the vertical diameter (implying that the cross section of the gaze cone is an oval). Horstmann and Linke (2021) have argued that the gaze cone is essentially the projection of the fovea, which is a roughly 5° large area where human daylight vision is very good (Kolb et al., 2020). This argument implies a circular shape of the gaze cone.

Technically speaking, the effect of picture size is essentially an effect of information loss due to either low screen resolution or low visual acuity. In the current Experiment 2, the effective screen resolution was 131 pixels per degree of visual angle. Thus, one pixel is somewhat less than 30 s of an arc which is below normal 20/20 Snellen acuity of 1 min of the arc. While the limiting factor in Experiment 2 was not the visibility of the pixels, pixilation would be expected to have similar effects. Ironically, while high visual definition and large picture size is preferable for many aspects of video communication, a loss of visual definition would not harm the impression of being looked at, but rather enhances it.

## Conclusion

In conclusion, we like to elaborate on some of the practical implications. If it is true that a somewhat upward directed gaze is perceived as direct (in particular, when the picture is positioned at the bottom of the screen), the optimal setting for a video-conference system is to use a camera near the bottom of the screen and present the speaker picture immediately above the camera in the center of the bottom row of tiles. As bottom cameras are rare (and not optimal for practical and aesthetical reasons), the second best recommendation for a top camera setting is to present the tile of the speaker (looker) in the upper center of the screen right below the fringe of the monitor. It is not advisable to use laptop computers as displays because the line of gaze is almost inevitably downward unless the position of the laptop computer is mounted on a socket. It might be useful to lure also the speaker to look at the center top row position. The simplest strategy, then, would be to dynamically relocate the speaker to the center top row position for all participants, including the speaker. Using small tiles also seems to be advisable. Small tiles tend to enlarge the gaze cone of the person on the tile, and they increase the probability that gaze is within the perceiver’s gaze cone when the looker looks at the tile nearest to the camera.

## Data Availability

The materials of the experiments and the datasets generated and/or analyzed during the current study are not publicly available due insufficient resources but are available from the corresponding author on reasonable request.
